# The Value of Brain Resting-State Functional Magnetic Resonance Imaging on Image Registration Algorithm in Analyzing Abnormal Changes of Neuronal Activity in Patients with Type 2 Diabetes

**DOI:** 10.1155/2021/6951755

**Published:** 2021-08-13

**Authors:** Jiajie Tong, Chunhui Shan, Congcong Hu

**Affiliations:** Department of Medical Imaging, Hebei Provincial People's Hospital, Shijiazhuang, Hebei 050051, China

## Abstract

The aim of this paper was to analyze the application value of resting-state functional magnetic resonance imaging (FMRI) parameters and rigid transformation algorithm in patients with type 2 diabetes (T2DM), which could provide a theoretical basis for the registration application of FMRI. 107 patients confirmed pathologically as T2DM and 51 community medical healthy volunteers were selected and divided into an experimental group and a control group, respectively. Besides, all the subjects were scanned with FMRI. Then, the rigid transformation-principal axis algorithm (RT-PAA), Levenberg–Marquardt iterative closest point (LMICP), and Demons algorithm were applied to magnetic resonance image registration. It was found that RT-PAA was superior to LMICP and Demons in image registration. The amplitude of low-frequency fluctuation (ALFF) values of the left middle temporal gyrus, right middle temporal gyrus, left fusiform gyrus, right inferior occipital gyrus, and left middle occipital gyrus in patients from the experimental group were lower than those of the control group (*P* < 0.05). The Montreal cognitive assessment (MoCA) score was extremely negatively correlated with the ALFF of the left middle temporal gyrus (*r* = −0.451 and *P* < 0.001) and highly positively associated with the ALFF of the right posterior cerebellar lobe (*r* = −0.484 and *P* < 0.001). In addition, the MoCA score of patients had a dramatically negative correlation with the ALFF of the left middle temporal gyrus (*r* = −0.602 and *P* < 0.001) and had a greatly positive correlation with the ALFF of the right posterior cerebellar lobe (*r* = −0.516 and *P* < 0.001). The results showed that RT-PAA based on rigid transformation in this study had a good registration effect on magnetic resonance images. Compared with healthy volunteers, the left middle temporal gyrus, right middle temporal gyrus, left fusiform gyrus, right inferior occipital gyrus, and left middle occipital gyrus in patients with T2DM showed abnormal neuronal changes and reduced cognitive function.

## 1. Introduction

T2DM often occurs in adults (especially obese patients) after the age of 35, accounting for more than 90% of diabetic patients [1]. At present, the etiology of T2DM is still insufficiently understood. It is generally believed that it may be the result of the combined effect on complex genetic and environmental factors. Factors such as high glucose toxicity, insulin resistance, susceptibility genes, and dyslipidemia may all participate in the pathogenesis [2, 3]. The disease is extremely harmful to the human. In severe cases, it could cause chronic complications of the whole body including nephropathy, retinopathy, neuropathy, macrovascular disease, microangiopathy, and diabetes foot, so as to result in a decrease in the patients' life quality [4]. The relevant data show that about 15% of patients with T2DM will eventually develop mild cognitive disorder or even dementia. Therefore, the timely diagnosis of cognitive disorder caused by T2DM has a great significance for the treatment and rehabilitation of patients.

Due to the development of medical imaging technology, MRI has been extensively used in brain examinations of patients with T2DM. The experimental design and related equipment of resting-state MRI are very simple. It only requires the patient to be quiet in an MRI machine, which can clearly show the subtle structure of the brain and reflect the electrical activity signals of several brain neurons, with the advantages of nontraumatic, simple operation, and easy cooperation [5]. Brown et al. [6] applied cardiac and abdominal MRI to dynamically monitor blood pressure and blood lipid levels of patients with T2DM and found that MRI could determine whether drug treatment could improve the left ventricular mass in patients with diabetes, so as to strengthen their cardiac protective effect. 3.0 MRI was employed to analyze the differences between the effects of high-intensity interval training and conventional treatment in patients with T2DM. Thus, the results showed that high-intensity interval training effectively promoted the quality and systolic function of the left ventricular wall in patients with T2DM, and its improvement was much greater than that of conventional treatment [7]. Image registration is the process of matching and superimposing two or more images acquired at different sensors (imaging equipment) or different times or under different conditions (weather, illuminance, camera position, and camera angle, etc.), and it has been widely applied to medical image processing, remote sensing data analysis, and computer vision [8]. However, it is always difficult to evaluate the registration results of the multimodal medical image since the medical image itself may have problems such as artifacts and noises. Therefore, how to select the method of MRI registration is a research direction currently.

In summary, 107 patients with T2DM and 51 healthy volunteers from the community physical examination were selected as EG and CG, respectively. Then, a new image registration algorithm RT-PAA was constructed based on RT and PAA with the simulation and comparison of LMICP and Demons algorithm, so as to record the ALFF values of each brain region of the two groups of subjects. Thus, the effects on image registration algorithm in clinical brain resting-state functional MR parameter diagnosis of T2DM patients can be comprehensively evaluated.

## 2. Materials and Methods

### 2.1. Sample Selection

107 patients with T2DM, who were pathologically diagnosed in the hospital from January 1^st^, 2018, to January 31^st^, 2020, were selected as EG, while 51 healthy volunteers from the community physical examination were selected as CG. All subjects were right-handed. The study had been approved by the Medical Ethics Committee of the hospital. The family members of the patient had understood the study and signed an informed consent.

The criteria for the samples inclusion contained that the patients did not use insulin sensitizers, were over 40 years old, had clear consciousness, were able to cooperate with the medical staff to have the examination, and had complete clinical medical records, images, and other materials.

The criteria for the samples exclusion included that patients had a history of stroke disease, took the drugs for improving cognition, had psychiatric diseases such as Parkinson's and epilepsy, suffered from the contraindication of MR scanning, and had other severe heart, lung, and kidney diseases.

### 2.2. Magnetic Resonance Imaging Registration Algorithm Based on Rigid Transformation

**Figure 1:**
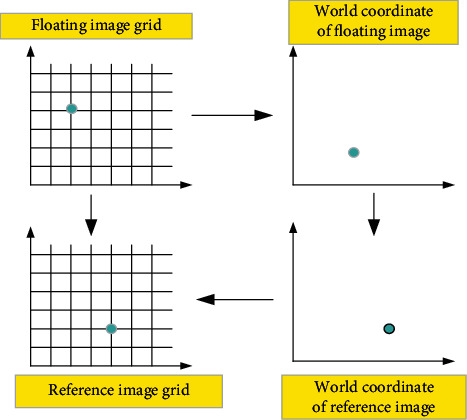
Transformation of medical image registration.

In the medical image registration, the corresponding anatomical points of reference image and floating image were generally matched through a spatial transformation to achieve the complete consistency of spatial positions. As shown in Figure 1, one point of the floating image could be aligned with the point of the reference image by space coordinate transformation, and the final result was that the points with diagnostic value were all overlapped.

In the actual MRI, the subject's head had no change in the relative position of the structure. When the reference image and the floating image were applied to the head registration, 6 parameters of the rigid change had to first be calculated, so the rigid variable vector could be expressed as follows:

(1) q⟶=l1,l,2l3,m4,m5,m6.

In equation (1), *l*_1_, *l*2, and *l*_3_ represented the translation distances of the brain on the coordinate axes *x*, *y*, and *z*, respectively, and *m*_4_, *m*_5_, and *m*_6_ represented the rotation angles on the coordinate axes *x*, *y*, and *z*, respectively. Then, a certain point *x*(*x*_1_, *x*_2_, *x*_3_) was translated to the point *y*(*y*_1_, *y*_2_, *y*_3_) along the translation vector *q*_*l*_(*l*_1_, *l*_2_, *l*_3_) based on Cartesian coordinates [9], and the relations among them could be expressed in the following equation:

(2) $$\begin{array}{c} y=x+q. \end{array}$$

Equation (2) was changed into a matrix form as follows:

(3) $$\begin{array}{c} \left[\begin{array}{c} y_{1}\\ y_{2}\\ y_{3}\\ y_{4} \end{array}\right]=\left[\begin{array}{cccc} 1 & 0 & 0 & l_{1}\\ 0 & 1 & 0 & l_{2}\\ 0 & 0 & 1 & l_{3}\\ 0 & 0 & 0 & l_{4} \end{array}\right]\left[\begin{array}{c} x_{1}\\ x_{2}\\ x_{3}\\ x_{4} \end{array}\right]. \end{array}$$

In equation (3), $I=\left[\begin{array}{cccc} 1 & 0 & 0 & l_{1}\\ 0 & 1 & 0 & l_{2}\\ 0 & 0 & 1 & l_{3}\\ 0 & 0 & 0 & l_{4} \end{array}\right]$ expressed translation transformation matrix. In addition, *x*(*x*_1_, *x*_2_, *x*_3_) rotated the angle of *m*_4_ based on coordinate axes *x*, which could be expressed in the following equation:

(4) $$\begin{array}{c} \left[\begin{array}{c} y_{1}\\ y_{2}\\ y_{3}\\ 1 \end{array}\right]=\left[\begin{array}{cccc} 1 & 0 & 0 & 0\\ 0 & cos\left(m_{4}\right) & sin\left(m_{4}\right) & 0\\ 0 & -sin\left(m_{4}\right) & cos\left(m_{4}\right) & 0\\ 0 & 0 & 0 & 1 \end{array}\right]\left[\begin{array}{c} x_{1}\\ x_{2}\\ x_{3}\\ 1 \end{array}\right]. \end{array}$$

In equation (4), $R_{x}=\left[\begin{array}{cccc} 1 & 0 & 0 & 0\\ 0 & cos\left(m_{4}\right) & sin\left(m_{4}\right) & 0\\ 0 & -sin\left(m_{4}\right) & cos\left(m_{4}\right) & 0\\ 0 & 0 & 0 & 1 \end{array}\right]$ represented rotation transformation matrix around coordinate axes *x*; furthermore, the following equations represent the rotation transformation matrixes around coordinate axes *y* and *z*, respectively.

(5) $$\begin{array}{c} M_{y}=\left[\begin{array}{cccc} cos\left(m_{5}\right) & 0 & sin\left(m_{5}\right) & 0\\ 0 & 1 & 0 & 0\\ -sin\left(m_{5}\right) & 0 & cos\left(m_{5}\right) & 0\\ 0 & 0 & 0 & 1 \end{array}\right], \end{array}$$

(6) $$\begin{array}{c} M_{z}=\left[\begin{array}{cccc} cos\left(m_{6}\right) & sin\left(m_{6}\right) & 0 & 0\\ -sin\left(m_{6}\right) & cos\left(m_{6}\right) & 0 & 0\\ 0 & 0 & 1 & 0\\ 0 & 0 & 0 & 1 \end{array}\right]. \end{array}$$

In equations (5) and (6), *M*_*y*_, *M*_*z*_ stood for the rotation transformation matrixes around coordinate axes *x* and *y*, respectively. Thus, the overall rotation transformation matrix was shown as follows:

(7) $$\begin{array}{c} M=M_{x}\cdot M_{y}\cdot M_{z}. \end{array}$$

Equation (8) was deduced based on the above.

(8) $$\begin{array}{c} L_{r}=L\cdot M_{x}\cdot M_{y}\cdot M_{z}. \end{array}$$

In equations (7) and (8), *M* stood for the rotation angle of the brain in space and *L* stood for the translation distance of the brain in space. Therefore, equation (9) expressed the behavioral matrix.

(9) $$\begin{array}{c} \left[\begin{array}{c} y_{1}\\ y_{2}\\ y_{3}\\ 1 \end{array}\right]=\left[\begin{array}{cccc} l_{11} & l_{12} & l_{13} & l_{14}\\ l_{21} & l_{22} & l_{23} & l_{24}\\ l_{31} & l_{32} & l_{33} & l_{34}\\ 0 & 0 & 0 & 1 \end{array}\right]\left[\begin{array}{c} x_{1}\\ x_{2}\\ x_{3}\\ 1 \end{array}\right]. \end{array}$$

To prevent local convergence from causing the problem that the parameters could not be optimized, PAA [10] was applied to limit the position deviation in a relatively small range, and the centroid coordinates in the Cartesian coordinate system should be calculated first through the following equations:

(10) $$\begin{array}{c} x_{c}=\frac{\sum x\cdot p\left(x,y,z\right)}{\sum p\left(x,y,z\right)}, \end{array}$$

(11) $$\begin{array}{c} y_{c}=\frac{\sum y\cdot p\left(x,y,z\right)}{\sum p\left(x,y,z\right)}, \end{array}$$

(12) $$\begin{array}{c} z_{c}=\frac{\sum z\cdot p\left(x,y,z\right)}{\sum p\left(x,y,z\right)}. \end{array}$$

In equations (10)–(12), *x*, *y*, and *z* represented coordinates of voxels and *p*(*x*, *y*, *z*) represented the grey values of the corresponding voxels. Then, the calculation equation of inertia matrix could be obtained.

(13) $$\begin{array}{c} H=\left[\begin{array}{ccc} H_{xy} & -H_{xy} & H_{xz}\\ -H_{yx} & H_{yy} & -H_{yz}\\ -H_{zx} & -H_{zy} & H_{zz} \end{array}\right]. \end{array}$$

In equation (13), *H* expressed the inertia matrix, which was standardized to let its feature vector be equal to the rotation matrix *M*. The calculation was as follows:

(14) $$\begin{array}{c} E=\left[\begin{array}{ccc} e_{11} & e_{12} & e_{13}\\ e_{21} & e_{22} & e_{23}\\ e_{13} & e_{23} & e_{33} \end{array}\right], \end{array}$$

(15) $$\begin{array}{c} M=M_{\alpha }M_{\beta }M_{\gamma }=\left[\begin{array}{ccc} 1 & 0 & 0\\ 0 & \cos\;\alpha & sin\alpha \\ 0 & -sin\alpha & \cos\;\alpha \end{array}\right]\left[\begin{array}{ccc} \cos\;\beta & 0 & sin\beta \\ 0 & 1 & 0\\ sin\beta & 0 & \cos\;\beta \end{array}\right]\left[\begin{array}{ccc} \cos\;\gamma & sin\gamma & 0\\ -sin\gamma & \cos\;\gamma & 0\\ 0 & 0 & 1 \end{array}\right]. \end{array}$$

In equation (15), *α*, *β*, and *γ* stood for the angles of the images around the axes *x*, *y*, and *z*, respectively; besides, *α*=arcsin(−*e*_32_/cos  *β*), *β*=arcsin  *e*_31_, and *γ*=arcsin(−*e*_31_/cos  *β*). The transformation parameters such as centroid and principal axis direction could be obtained from the above to carry out the image registration, and the result of the image registration was set as RT-PAA algorithm based on RT and PAA.

### 2.3. Algorithm Simulation Experiment

The dataset from the Wellcome Trust Centre for Neuroimaging was selected as samples in the study. This dataset identified activation regions corresponding to brain functions through different auditory stimuli, including 95 functional imaging data. LMICP algorithm based on contour feature [11] was introduced to compare with Demons algorithm under multimodal elastic registration [12]. The image registration parameters of the three algorithms were recorded including translation in the *X* direction, translation in the *Y* direction, rotation angle, and iteration times.

### 2.4. Resting-State Magnetic Resonance Scanning

The 3.0T MR scanner produced by Siemens (Germany) was employed to detect the subjects. When the subject was scanned, he or she should lie flat in the MRI machine, keep the head and body still, try to stay awake, use special earplugs to shield the noise, and locate the center of the scan on the center of the eyebrow. Requirements for the scanning parameters included gradient plane echo sequence and included that TR equaled 2,500 milliseconds, TE equaled 120 milliseconds, layer spacing was 1.5 mm, layer thickness was 5 mm, matrix equaled 521 multiplied by 521, and field of view equaled 250 × 250 mm^2^. After the scanning was completed, the image data were transmitted to the matrix laboratory (MATLAB R2015b) platform for processing and calculation, so as to obtain the ALFF values of each brain area.

### 2.5. Clinical Data Collection

The age, height, weight, gender, triglycerides, systolic blood pressure (SBP), total cholesterol, and diastolic blood pressure (DBP) of the subjects in the two groups were recorded. The subjects were assessed by MoCA. What is more, the patient's normal MR and resting-state MR images were also recorded.

### 2.6. Statistical Method

The data processing of the study was analyzed by SPSS19.0 version statistical software, and the mean ± standard deviation $\left(\bar{x}\pm s\right)$ and the percentage (%) were applied to the measurement data and count data, respectively. The paired *t*-test was adopted to compare the translation amount-*X;* translation amount-*Y*; rotation angle; and iteration times of the RT-PAA, LMICP, and Demons algorithm. Comparison on the ALLF values of LMTG, right middle temporal gyrus (RMTG), left fusiform gyrus (LFG), right inferior occipital gyrus (RIOG), left middle occipital gyrus (LMOG), RPLC, and RC was analyzed by variance. There was a difference with statistical meaning at *P* < 0.05.

## 3. Results

### 3.1. Simulation Experiment of Algorithm Registration Performance

**Figure 2:**
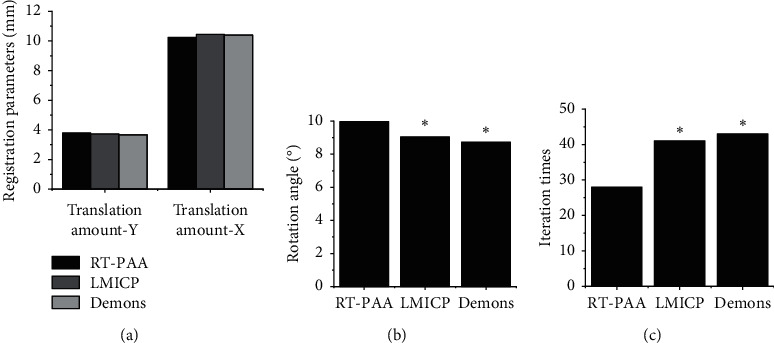
Comparison on image registration parameters of different algorithms. (a) Comparison on translation amount-*X* and translation amount-*Y* among RT-PAA, LMICP, and Demons algorithm. (b) Rotation angles of the three algorithms. (c) Iteration times of the three algorithms. ^∗^expressed that there was a statistical difference compared to RT-PAA algorithm (*P* < 0.05).

**Figure 3:**
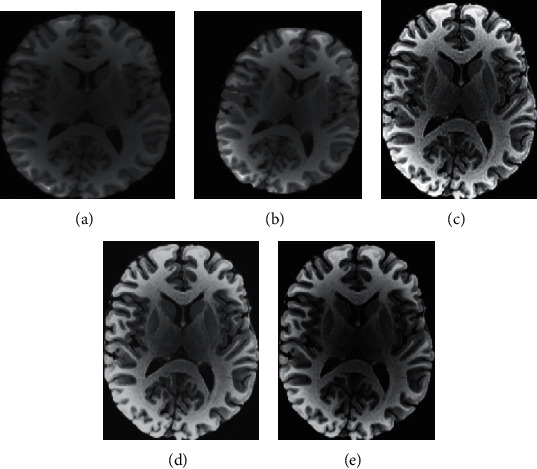
Comparison on image registration effects of different algorithms. (a, b) Reference image and preprocessed image to be registered, respectively. (c–e) The RT-PAA, LMICP, and Demons algorithm registration images, respectively.

As shown in Figure 2, there was no statistical difference among the RT-PAA, LMICP, and Demons algorithm in the image registration parameters translation amount-*X* and translation amount-*Y*(*P* > 0.05). The rotation angles of the image registration parameters in the RT-PAA algorithm were greatly bigger than the angles of the LMICP and Demons algorithm, showing a statistical meaning (*P* < 0.05). However, iteration times of the image registration parameters in the RT-PAA algorithm were hugely less than the times of LMICP and Demons algorithm, with a statistically significant difference (*P* < 0.05). In Figure 3, Figures 3(a) and 3(b) show the reference image and preprocessed image to be registered, respectively, and Figures 3(c)–3(e) show the RT-PAA, LMICP, and Demons algorithm registration images, respectively. It showed that it had a higher degree of display clarity and coincidence, and its registration effect was the best.

### 3.2. Comparison of Basic Data of the Subjects in the Two Groups

**Figure 4:**
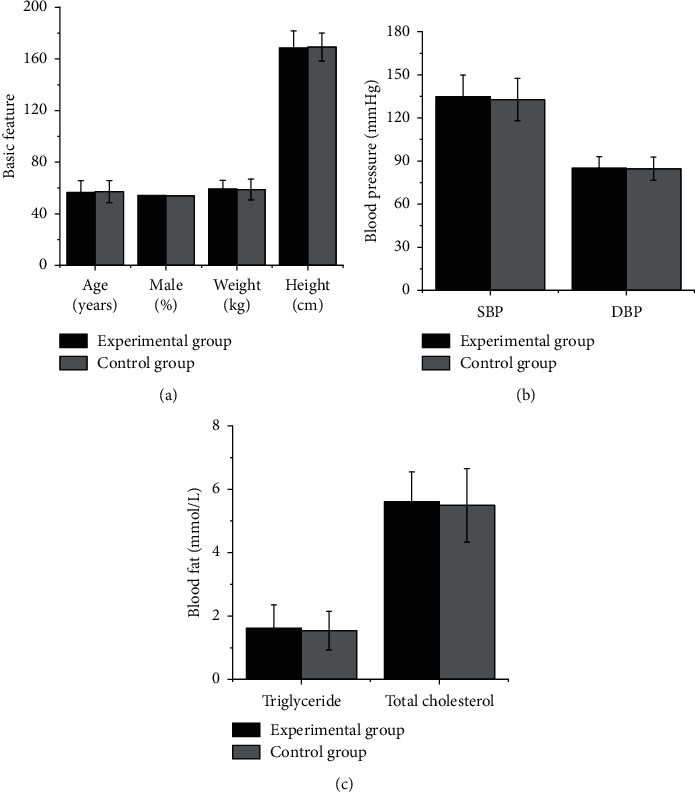
Comparison on basic data of the subjects in the two groups. (a) Comparison of age, height, weight, and the percentage of male in subjects from the EG and CG. (b) Comparison of SBP and DBP in subjects from the two groups. (c) Comparison of triglyceride and total cholesterol between the two groups.

**Figure 5:**
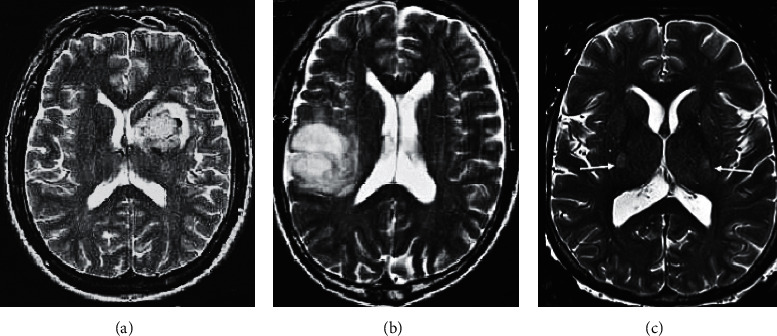
MRI images of some subjects. (a) MRI image of a female patient. (b) MRI image of a male patient. (c) MRI image of a patient in CG.

It is illustrated in Figure 4 that the differences in age, height, weight, gender, SBP, DBP, triglyceride, and total cholesterol among the subjects from EG and CG were not statistically meaningful (*P* > 0.05).  Figure 5 shows MRI images of some subjects.  Figure 5(a) shows that there was a characteristic bat wing-like lesion at the base of the pons, which was symmetrically distributed with *T*1 low signal and *T*2 high signal without enhancement effect.  Figure 5(b) illustrates that there were the paraventricular softening foci of the left lateral ventricle with glial hyperplasia; the tumor was small, while the edema area was large; the edema shape was finger-pressing; and there was the mass effect. Moreover, there was a normal brain tissue without any lesions in Figure 5(c).

### 3.3. Comparison of the Low-Frequency Fluctuation Amplitude Values of the Remarkably Changeable Brain Regions of the Subjects in the Two Groups

**Figure 6:**
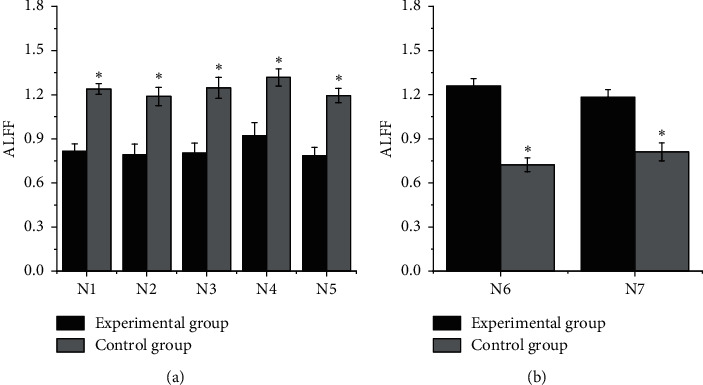
Comparison on ALFF values of the remarkably changeable brain regions of the subjects in the two groups. (a) Comparison on the ALFF values of LMTG, RMTG, LFG, RIOG, and LMOG in subjects from EG and CG. (b) Comparison on the ALFF values of RPLC and RC in subjects from the two groups. ∗ represented that the difference had the statistical meaning by comparing with the case before treatment (*P* < 0.05).

**Figure 7:**
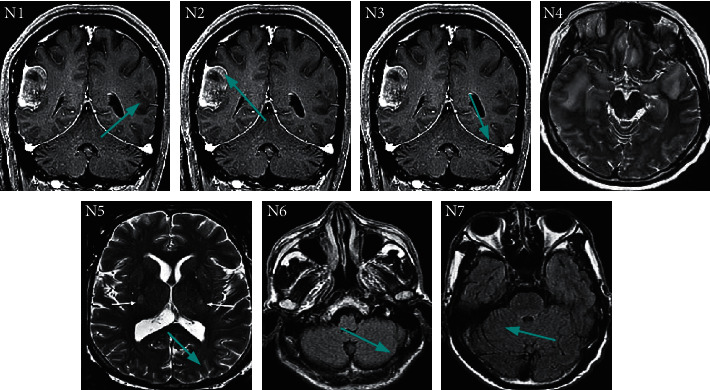
The brain region images of the remarkably changeable ALFF values of one patient (The green arrows represented the brain regions.).

As shown in Figures 6 and 7, *N*1, *N*2, *N*3, *N*4, *N*5, *N*6, and *N*7 expressed the LMTG, RMTG, LFG, RIOG, LMOG, RPLC, and RC, respectively. It indicated that the ALFF values of LMTG, RMTG, LFG, RIOG, and LMOG of the subjects in EG were substantially lower than the values of CG. The ALFF values of RPLC and RC were higher than those of CG, with the statistically significant difference (*P* < 0.05).

### 3.4. Comparison of the Scale Scores of the Subjects in the Two Groups

**Figure 8:**
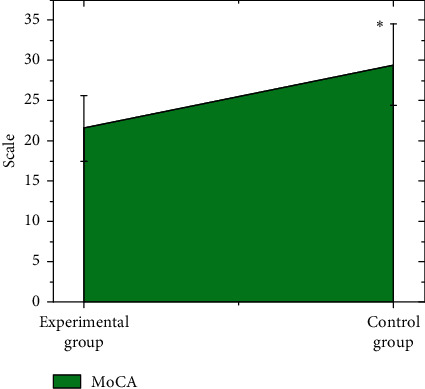
Comparison on the scale scores of the subjects in both groups. ∗ expressed that the difference had the statistical meaning by comparing with EG (*P* < 0.05).

It is shown in Figure 8 that the MoCA score of *v* was 21.56 ± 4.11, while the MoCA score of CG was 29.47 ± 5.08. In addition, the MoCA scores of EG were enormously lower than the scores of CG, indicating a statistical difference (*P* < 0.05).

### 3.5. Correlation Analysis of the Low-Frequency Fluctuation Amplitude Value and Scale Score in the Patients from the Experimental Group

**Figure 9:**
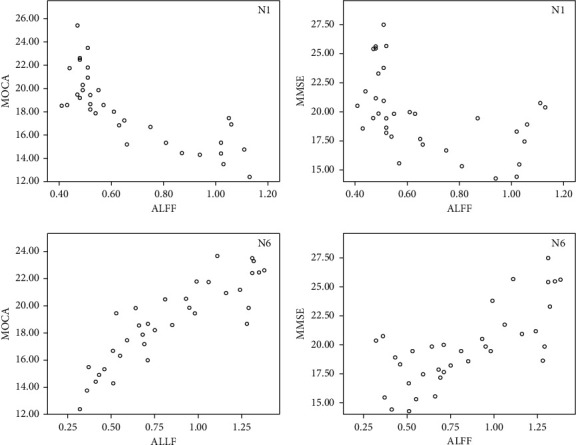
Scatter plot of the correlation between the patient's ALFF value and scale scores. (a, b) The correlation scatter diagram between ALFF and MoCA. (c, d) The correlation scatter diagram between ALLF and MoCA.

As shown in Table 1, the ALFF value of the patient's LMTG had an extremely negative correlation with the MoCA score (*P* < 0.001), but there was a markedly positive correlation of the ALFF value and MoCA score in the patient's posterior cerebellar lobe (*P* < 0.001). Besides, there are four scatter plots of correlation between the patient's ALFF value and scale scores for N1 and N6 in Figure 9.

## 4. Discussion

Clinically, the imaging results of multiple modes in the same patient are usually combined to improve the medical diagnosis and treatment, which involves the image registration problem [13]. A new image registration algorithm RT-PAA was constructed based on RT and PAA and compared with LMICP and Demons algorithm. The results showed that the rotation angle of RT-PAA algorithm was extremely higher than that of LMICP and Demons, and the iteration times were hugely lower than the times of LMICP and Demons algorithm (*P* < 0.05), which was similar with the research results of Stephen et al. [14], so as to indicate that RT-PAA had the best image quality of registration and had higher accuracy and faster speed. The ALFF values of LMTG, RMTG, LFG, RIOG, and LMOG in the patients from EG were greatly lower than the values of CG (*P* < 0.05), which was similar to the results of Aojula et al. [15]; it indicated that ALFF has been proved to reflect the intrinsic activity information of specific brain regions. Furthermore, the results of the study showed that the LMTG, RMTG, LFG, RIOG, and LMOG of patients with T2DM were abnormal. The ALFF values of RPLC and RC in patients from EG were higher than those of CG (*P* < 0.05), which was different from the research results of Ozaki et al. [16]. Therefore, the reason might be that the enhanced neural activity in the characteristic brain regions of patients might be additional neural activity compensation, which can be applied to make up for the reduced cognitive ability in other brain regions.

It was found from the scale evaluation that the MoCA score of EG were steeply lower than scores of CG with the statistical difference (*P* < 0.05), which was similar to the results of Alger et al. [17]. It indicated that there were multidimensional disorders of cognitive function, compared with T2DM patients. The ALFF value of the patients' LMTG was dramatically negatively correlated with MoCA score (*P* < 0.001), which showed that the decline of the patient's cognitive function may be caused by the abnormal ALFF of LMTG in the specific brain area. There was a substantially positive correlation of the ALFF value and MoCA score in the patient's RPLC (*P* < 0.001). Moreover, RPLC was the core brain area that regulated the emotional state and appetite of patients. Therefore, it showed that RPLC could play a compensatory role to prevent patients from the decline of cognitive function [18]. It is speculated that the resting-state MR parameter ALFF based on the image registration algorithm can reflect the spontaneous neuronal activity in a specific area of T2DM patients.

## 5. Conclusion

RT-PAA, a new image registration algorithm, was constructed based on the RT and PAA, which was simulated and compared with LMICP and Demons algorithm. Also, RT-PAA was used for the diagnosis of resting-state functional MRI in 107 patients with T2DM and 51 healthy volunteers. The results indicated that RT-PAA algorithm had the best quality of registration images and also had higher accuracy and faster speed, compared with the traditional algorithm. ALFF, a resting-state MR parameter based on RT-PAA algorithm, could reflect the abnormal activity of spontaneous neurons in specific regions in patients with T2DM. In addition, cognitive dysfunction was associated with ALFF in the LMTG and RPLC. However, only a few cases were included in this study, and no specific pathological classification was made for T2DM patients. Therefore, the sample size of patients should be increased for further follow-up. In conclusion, the results of the study provided a theoretical basis for image registration algorithm in MRI diagnosis of T2DM.

## Figures and Tables

**Table 1:** Correlation analysis of the ALFF value and scale score in the patients.

Brain regions	MoCA
	*r*
LMTG	−0.451^*∗∗*^
RMTG	−0.122
LFG	−0.244
RIOG	−0.185
LMOG	−0.261
RPLC	0.484^*∗∗*^
RC	0.275

^*∗∗*^, *P* < 0.001.

## Data Availability

The data used to support the findings of this study are available from the corresponding author upon request.
